# Child development, growth and microbiota: follow-up of a randomized education trial in Uganda

**DOI:** 10.7189/jogh-09-010431

**Published:** 2019-06

**Authors:** Prudence Atukunda, Grace K M Muhoozi, Tim J van den Broek, Remco Kort, Lien M Diep, Archileo N Kaaya, Per O Iversen, Ane C Westerberg

**Affiliations:** 1 Department of Nutrition, Institute of Basic Medical Sciences, University of Oslo, Oslo, Norway; 2 Department of Human Nutrition and Home Economics, Kyambogo University, Kampala, Uganda; 3 Netherlands Organization for Applied Scientific Research (TNO), Microbiology and Systems Biology, Zeist, the Netherlands; 4 Department of Molecular Cell Biology, VU University Amsterdam, the Netherlands; 5 Oslo Centre for Biostatistics and Epidemiology, Oslo University Hospital, Oslo, Norway; 6 School of Food Technology, Nutrition and Bioengineering, Makerere University, Kampala, Uganda; 7 Department of Haematology, Oslo University Hospital, Oslo, Norway; 8 Division of Human Nutrition, Stellenbosch University, Tygerberg, South Africa; 9 Institute of Health Sciences, Kristiania University College, Oslo, Norway; * Equal authorship

## Abstract

**Background:**

Undernutrition impairs child development outcomes and growth. In this follow-up study of an open cluster-randomized intervention trial we examined the effects of an education package delivered to mothers in rural Uganda on their children’s development, growth and gut microbiota at 36 months of age.

**Methods:**

The parental trial included 511 mother-child pairs recruited when the children were 6-8 months. In that trial, a nutrition, stimulation and hygiene education was delivered to mothers in the intervention group while the control group received routine health care. A follow-up sample of 155 pairs (intervention n = 77, control n = 78) were re-enrolled when the children were 24 months. Developmental outcomes were assessed with the Bayley Scales of Infant and Toddler Development (BSID-III) composite scores for cognitive (primary endpoint), language and motor development. Development outcomes were also evaluated using the Ages and Stages Questionnaire (ASQ) and the Mullen Scales of Early Learning (MSEL). Other outcomes included growth and gut microbiota composition.

**Results:**

The demographic characteristics were not different (*P* > 0.05) between the intervention and control groups and similar to those of the parental study. The intervention group had higher BSID-III scores than controls, with mean difference 10.13 (95% confidence interval (CI): 3.31-17.05, *P* = 0.002); 7.59 (1.62-13.66, *P* = 0.01); 9.00 (2.92-15.40, *P* = 0.005), for cognitive, language and motor composite scores, respectively. An improvement in the intervention compared to the control group was obtained for both the ASQ and the MSEL scores. The mean difference in height-for-age z-score was higher in the intervention compared to the control group: 0.50 (0.25-0.75, *P* = 0.0001). Gut microbiota composition did not differ significantly between the two study groups.

**Conclusions:**

The maternal education intervention had positive effects on child development and growth at three years, but did not alter gut microbiota composition. This intervention may be applicable in other low-resource settings.

**Trial registration:**

ClinicalTrials.gov registration number NCT02098031.

Undernutrition among children in developing countries is a major, global health challenge causing more than one-third of under-five deaths [1]. About 200 million children below five years worldwide who are undernourished do not meet their development potential [2]. In addition to stunting, poor cognitive stimulation, and adverse environmental conditions, low maternal education is recognized as risk factors for impaired child development [3]. In line with this, undernourished children are at risk of impaired structural development of the central nervous system (CNS) and extra- neuronal tissue [4]. Adequate childhood nutrition thus promotes healthy growth and development outcomes [5-7].

The underlying causes of chronic undernutrition are complex. In addition to inadequate food quantity and quality of food, a combination of poor sanitation and hygiene resulting in sustained exposure to enteric pathogens plays an important role [8]. Pregnancy and the first two years of life are important periods for interventions to improve child growth and cognitive development, and can be considered both a window of vulnerability as well as a window of opportunity [9]. The development of the gut microbiota is mostly accomplished within the first three years of life. Interventions directed towards an appropriate maturation of the gut microbiota and its associated metabolic potential, may support healthy growth and cognitive development [10]. In line with this, there are new insights into how the CNS and cognitive development may be influenced by the gut microbiota through the so-called “gut-brain axis” [11]. Moreover, a number of studies report systematic differences in the composition between rural Africa and urban Europe, indicating that the gut microbiota is tailored to local diet, specific nutritional requirements of the host and hygiene practices [12-14]. However, a possible role of gut microbiota in undernutrition and child development has not been adequately addressed. Inadequate caregiving skills and stimulation can also adversely impact development and growth of small children, in particular in low-resource settings. Integration of nutrition and stimulation in a Pakistani trial highlighted a potential for child development and linear growth benefits [5].

In 2013 we initiated a cluster-randomized controlled trial to examine the effect on growth and development of a 6-month intervention comprised of nutrition, stimulation and hygiene education among impoverished mothers of children aged 6-8 months in rural districts of Uganda [15]. The intervention consisted of educating mothers aimed at (i) increasing dietary diversity to improve nutrient intake as well as continued breastfeeding, (ii) improving hygiene and sanitation practices, and (iii) enhancing stimulation based on a social-cognitive learning theory to improve development. Whereas this intervention did not alter child growth at the age of 20-24 months, cognitive, language and motor development improved markedly [15]. In order to examine if these findings were sustainable over time, we decided to perform a follow-up study. Notably, a long term follow-up of such a nutrition education intervention has previously not been done in a resource-constrained setting as Uganda. We now examined development, growth and gut microbiota composition among a subsample of these children at the age of 36 months.

## METHODS

### Study design and approvals

This is a follow-up study of a two-armed, open cluster-randomized education intervention regarding nutrition, stimulation and hygiene among impoverished mothers of children aged 6-8 months in the Kisoro and Kabale districts of South-Western Uganda. Details of the intervention have recently been published [15]. All mothers gave written or thumb-printed, informed consent to participate and could decline an interview or assessment at any time. The study was approved by The AIDS Support Organisation Research Ethics Committee (No. TASOREC/06/15-UG-REC-009) and by the Uganda National Council for Science and Technology (No. UNCST HS 1809) as well as by the Norwegian Regional Committee for Medical and Health Research Ethics (No. 2013/1833). The trial was registered with clinicaltrials.gov (NCT02098031). We report the data according to the CONSORT guidelines.

### Randomisation of the parental and follow-up participants

For the parental trial we used proportionate sampling, 10 sub-counties (ie, clusters) were obtained (6 out of 19 in Kabale and 4 out of 14 in Kisoro) to participate in the study. We used a three-stage procedure to identify households for the study. First, by simple random sampling, three sub-counties in Kabale were allocated to the intervention group and the other three to the control group. Similarly, two sub-counties were allocated to the intervention and the other two to the control group in Kisoro district. Second, all the villages in each participating sub-county (intervention or control) were listed alphabetically and assigned numbers in an ascending order. By use of computer-generated random numbers, villages to whose assigned number matched with the random numbers were selected. The intervention villages did not share common geographical boundaries with control villages to minimize contamination of the intervention contents between the two study groups. Third, by complete enumeration, all consenting households with children aged 6-8 months within a participating village were recruited to the study. If a household had more than one eligible child, the youngest was selected, and in the case of twins, we randomly selected one for evaluation. We finally enrolled 511 mother-child pairs in the parental study and they were randomised to the intervention (n = 263) or the control (n = 248) group. The intervention group received the nutrition, hygiene and stimulation education in addition to routine health care while the control group received only routine health care.

The child had to be 20-24 months during the period of January-May 2015 to be included in the current follow-up study since age dynamic gut microbial shifts occur at this age resulting in an adult-like, stable composition [16], and developmental milestones at this age may predict IQ at 5-6 years when children are about to start school [17]. Based on a sample size calculation we then randomly selected participants from each of the two study groups (n = 77 from the intervention group and n = 78 from the control group). Data was collected when the children were 20-24 months and at 36 months. The data collection teams in the follow-up study were masked to group allocation and never had any interaction with the study team that delivered the education intervention in the parental trial.

### Contents of the education intervention in the parental trial

The intervention was conducted by the study team at three group meetings over a period of 6 months to 26 groups of mothers (6-10 mothers per group), and was detailed recently [15]. Briefly, it was delivered by a trained education team and included two behavior change techniques: providing information and prompt practice (ie, demonstrations of preparing food and stimulation of the children). The nutrition education curriculum was based on the 10 guiding principles of complementary feeding [18]. Recipes were formulated and cooking demonstrated using locally available foods with emphasis on protein. Moreover, the need to take ill children to hospital for medical attention and to increase the feeding frequency during and after illness was emphasized. Hand-washing before feeding as well as use of clean utensils during food preparation and feeding was part of the hygiene intervention. A novel aspect of this intervention was the focus on oral hygiene, and with distribution of tooth brushes to all household members and demonstration of their use. The education team highlighted the importance of play to improve cognitive, language and motor development. The stimulation intervention was based on social-cognitive learning theory [19]. In addition to the three group meetings, the women met at monthly intervals to practice what they had learnt and ensuring compliance to the intervention [15].

### Assessments of outcomes

The child development assessments were performed by three bachelor degree holders in psychology whereas two graduates of laboratory technology collected stool samples. Two bachelor degree holders in nutrition collected the anthropometric data. These three data collection teams participated in training sessions to ensure uniform and standardized procedures. Assessments were administered in the local language and conducted in hired, secluded rooms in the villages without interruptions to minimize distractions. To promote reliability, the child development assessments were administered first, followed by anthropometric measurements, stool sampling and then interviews with the mothers.

The Bayley Scales of Infant and Toddler Development-III (BSID-III), the Ages and Stages Questionnaire (ASQ) and the Mullen Scales of Early Learning (MSEL; Supplementary information) were used [15]. The BSID-III scale is known to be the most comprehensive child development measure for children up to 3.5 years and has been adapted and used in similar settings [20]. The ASQ is a parent/caregiver completed screening scale with excellent psychometric properties which capture and establish a wide range of adaptive behaviors, and previously used in this setting [21]. Both tools were used because we did not include the social-emotional scale of BSID-III. The BSID-III and the ASQ were administered at 20-24 and at 36 months. MSEL was introduced at 36 months to assess early intellectual development and readiness for school, and it has been validated for use in rural Uganda [22]. Inter-observation agreement between the child assessment team was good indicated by an intra-class correlation coefficient (ICC) of 0.75 (*P* = 0.0001) for BSI-III, 0.79 (*P* = 0.0001) for ASQ and 0.77 (*P* < 0.001) for MSEL.

Weight, height, and head circumference (HC) were measured as recommended by WHO [21], with a Seca-scale model 881 (Hamburg, Germany) to the nearest 0.1 kg. Height was measured (to the nearest 0.1 cm) with a Seca board (SO114530). HC was measured with a non-stretchable tape (Seca, S0145630 PAC-50). Anthropometric data were converted to z-scores, height-for-age (HAZ), weight-for-age (WAZ), weight-for-height (WHZ), and head circumference (HCZ), using the Anthro (version 3.2.2) software, a nutritional assessment tool based on WHO standards. A z-score<-2 SD from the median of the WHO reference standards indicated stunting for HAZ, underweight for WAZ and wasting for WHZ, respectively [23].

We collected stool samples using sterile cotton swabs (COPAN Diagnostics Inc, Murrieta, CA) and frozen at -20°C within 24 hours of collection. The samples were then air-dried and shipped to the Netherlands for further processing and analyses (Supplementary information). These storage conditions have a very limited effect on the microbial composition [24]. All 16S rRNA amplicon paired end reads (n = 560) of the gut microbiota samples sequenced in this study can be accessed at Sequence Read Archive (SRA) SUB4476421.

### Statistical analyses

The primary outcome in the current follow-up study was cognitive development assessed with the BSID-III at 36 months. Previous intervention studies in similar low-resource-settings report a mean difference of about 0.5 SD in child development score between intervention and control groups [5,25]. To detect a difference between the two study groups in the BSID-III cognitive composite score at 36 months of 0.5 SD (corresponding to 7.5 points) with a power of 0.8 and α of 0.05, 63 children per group was required. To account for an intra-cluster correlation of 0.01 and dropouts, the mean number of children per sub-county was 15, thus a total of 155 children were included [15,26]. Among these 155 we randomly selected the 77 children from the intervention group and the 78 children from the control group at 20-24 months. Child development outcomes and growth were analyzed using Stata/SE (StataCorp. 2015, Stata Statistical Software: Release 14. College Station, Stockholm, Sweden) and SPSS version 22.0 (IBM SPSS Statistics, IBM Corp., Armonk, NY). Significance level was set at *P* < 0.05. We used a mixed effect linear regression to compare the intervention with the control group and estimated ICC. Differences between the two study groups are given as mean (SD or 95% CI).

All statistical analysis of gut microbiota on the 16S rRNA amplicon sequencing data was performed using R version 3.3.2 (R Core Team, 2016) [27]. The 16S rRNA amplicon sequencing data was rescaled and transformed using Wisconsin double and square root transformations. The PERMANOVA procedures, Shannon and 1-Simpson’s diversity indices were performed as implemented in the ‘vegan’ package [28]. Whereas increasing values for the Shannon diversity index indicate more diversity, the opposite is true for the 1-Simpson’s index. All PERMANOVA analyses were performed using the Bray-Curtis distance measure. All phyla and genera were included in the statistical analysis.

## RESULTS

### Study participants

**Figure 1:**
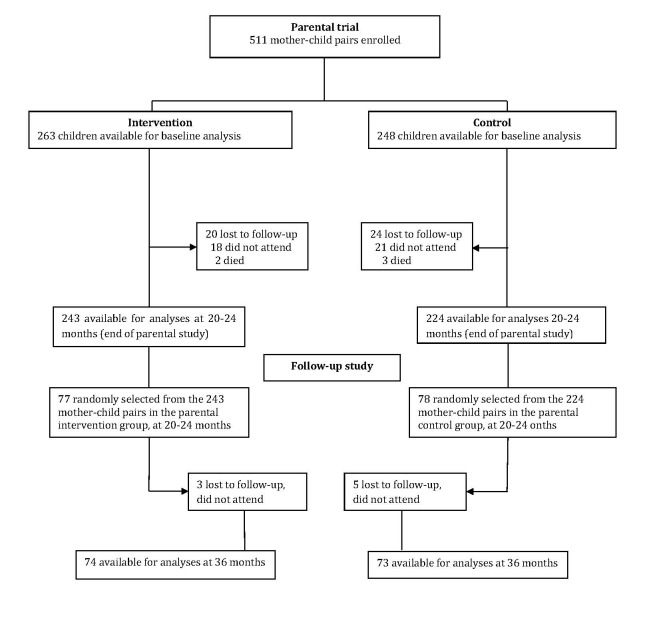
Profile of the parental trial and the follow-up study.

One hundred and fifty-five mother-child pairs were included at 20-24 months (**Figure 1**). By 36 months, eight of them were lost to follow-up (three in the intervention group and five in the control group). There were no significant differences in the characteristics between the parental cohort (data obtained at baseline) and the follow-up cohort (data obtained at 20-24 months; **Table 1**), thus no adjustments for baseline differences were made in subsequent analyses.

**Table 1:** Study population characteristics for the parental trial at baseline and at start of the follow-up study*

Characteristics	Parental trial (data obtained at baseline)		Follow-up study (data obtained at 20-24 months)		*P*-value
	**Intervention (n = 263)**	**Control (n = 248)**	**Intervention (n = 77)**	**Control (n = 78)**	
**Children (*n*, %):**					
Males	139 (52.9)	123 (49.6)	44 (57.1)	41 (52.6)	0.75
Females	124 (47.1)	125 (50.4)	33 (42.9)	37 (47.4)	0.40
Age at inclusion (months)	7.4 (0.8)	7.3 (0.9)	21.4 (1.0)	21.2 (1.0)	0.24
Stunting†	55 (20.9)	70 (28.0)	32 (18.1)	46 (36.6)	0.06
Underweight†	25 (9.5)	36 (14.5)	6 (8.3)	8 (11.3)	0.37
Wasting†	12 (4.6)	12 (4.8)	3 (4.2)	2 (2.8)	0.50
**BSID-III composite score:**					
Cognitive	114.9 (21.3)	99.3 (17.1)	116.1 (15.6)	105.9 (15.9)	0.89
Language	98.3 (14.3)	88.4 (9.1)	106.5 (14.8)	98.9 (12.8)	0.50
Motor	113.7 (18.9)	99.1 (14.3)	122.3 (18.7)	113.3 (19.9)	0.49
**ASQ scores:**					
Communication	40.8 (14.5)	33.8 (15.3)	51.4 (9.9)	48.1 (11.4)	0.36
Gross motor	52.8 (10.3)	46.9 (13.8)	55.6 (7.0)	53.8 (9.7)	0.51
Fine motor	44.6 (9.9)	40.4 (11.5)	47.9 (10.8)	42.5 (13.9)	0.23
Problem solving	49.5 (11.7)	40.6 (13.1)	44.0 (12.3)	40.1 (12.7)	0.24
Personal-social	41.0 (11.3)	36.6 (11.1)	48.7 (10.8)	45.8 (9.9)	0.35
**Illness at study time (*n*, %):**					
Yes	94 (35.7)	71 (28.6)	47 (61.0)	40 (51.3)	0.21
No	169 (64.3)	177 (71.4)	30 (39.0)	38 (48.7)	0.38
**Maternal data:**					
Maternal education (years)	4.9 (2.8)	4.9 (2.8)	5.5 (2.5)	5.0 (2.6)	0.20
Maternal age (years)	26.1 (5.8)	26.8 (6.3)	26.2 (6.1)	27.4 (6.4)	0.27
Number of children per mother	3.4 (2.2)	3.3 (2.2)	3.4 (2.2)	3.3 (2.2)	0.25
**Household data:**					
Household head age (years)	31.3 (7.7)	32.6 (19.4)	30.2 (7.3)	33.1 (10.9)	0.06
Household head education (years)	6.4 (3.1)	5.9 (3.1)	6.6 (3.3)	6.5 (3.4)	0.29
Household size (n)	5.5 (2.1)	5.5 (2.1)	5.7 (2.2)	5.8 (2.2)	0.76
Household poverty score	47.8 (11.7)	47.6 (11.4)	49.0 (11.6)	46.3 (12.3)	0.18
Sanitation composite score	7.2 (1.9)	7.3 (1.9)	7.0 (1.8)	7.1 (1.9)	0.83

ASQ – Ages and Stages Questionnaire, BSID – Bayley’s Scales of Infant and Toddler Development

*Values are means (SD) unless otherwise stated.

†*z-* score values are<-2SD of the median of the reference population.

### Development outcomes

Overall, the intervention significantly improved all child development outcomes (ie, cognitive, language and motor composite scores) based on the BSID-III at 36 months (**Table 2**). The Cohen’s d effect sizes at 36 months were medium (cognitive 0.57, language 0.56 and motor 0.50). The effect of the intervention on the ASQ mean scores for communication, gross motor, problem solving, and personal social development, was significantly higher in the intervention group compared with the controls at 24 months (**Table 3**). At 36 months, the ASQ fine motor scores were significantly higher in the intervention group compared with the controls. The Cohen’s d effect sizes at 36 months ranged from small to medium for the ASQ scores (gross motor 0.16, personal social development 0.25, problem solving 0.29, fine motor 0.49 and communication 0.68). Also, the MSEL fine motor, language (receptive and expressive), cognitive and early learning composite standard scores were significantly higher in the intervention compared to the controls at 36 months (**Table 4**). In contrast, the MSEL visual reception scores were not different between the two study groups. The corresponding mean Cohen’s d effect sizes were: 0.23, 0.44, 0.34, 0.42, 0.42, and 0.36 for MSEL visual reception, fine motor, receptive language, expressive language, cognitive total score and early learning score, respectively.

**Table 2:** Composite scores derived from the Bayley Scales of Infant and Toddler Development-III scales*

Age of child (months)	Intervention (n = 73-77)†	Control (n = 74-78)†	Between group difference‡	*P-*value§	ICC
**Cognitive composite scores:**			
20-24	117.84 (20.86)	101.58 (19.14)	16.26 (9.57 to 23.04)	0.0001	0.05
36	116.07 (15.55)	105.94 (15.99)	10.13 (3.31 to 17.05)	0.002	
**Language composite scores:**					
20-24	100.31 (12.91)	89.00 (9.32)	11.31 (5.43 to 17.28)	0.0001	0.06
36	106.54 (14.79)	98.95 (12.77)	7.59 (1.62 to 13.66)	0.010	
**Motor composite scores:**					
20-24	113.79 (16.06)	100.04 (15.47)	13.75 (7.80 to 20.01)	0.0001	0.01
36	122.32 (18.74)	113.32 (19.89)	9.00 (2.92 to 15.40)	0.005	

ICC – intra-class correlation coefficient

*Values are means (standard deviation) unless otherwise stated.

**†**The variation in n was due to missing data because some children did not complete all the tests.

‡Mean differences (95% CI) of Bayley Scales of Infant and Toddler Development-III composite scores.

§*P-*value is for the difference between the two study groups adjusted for clusters.

**Table 3:** Mean scores from the Ages and Stages Questionnaire*

Age of child (months)	Intervention (n = 71-74)†	Control (n = 70-73)†	Between group difference‡	*P*-value§	ICC
**Communication scores:**					
20-24	41.37 (14.04)	31.58 (18.45)	9.79 (3.90 to 15.76)	0.001	0.06
36	51.41 (9.96)	48.11 (11.40)	3.30 (-2.68 to 9.33)	0.28	
**Gross motor scores:**					
20-24	53.46 (10.76)	46.47 (15.79)	6.99 (2.47 to 11.60)	0.003	0.00
36	55.58 (7.04)	53.80 (9.72)	1.78 (-2.80 to 6.47)	0.44	
**Fine motor scores:**					
20-24	45.73 (9.93)	42.04 (12.58)	3.69 (-0.27 to 8.01)	0.067	0.07
36	47.93 (10.80)	42.52 (13.94)	5.41 (1.36 to 9.81)	0.010	
**Problem solving scores:**					
20-24	50.35 (10.19)	38.94 (14.24)	11.41 (7.24 to 15.57)	0.0001	0.02
36	44.02 (12.25)	40.06 (12.69)	3.96 (-0.31 to 8.21)	0.069	
**Personal-social development scores:**					
20-24	43.24 (10.41)	36.81(10.05)	6.43 (1.99 to 10.85)	0.0001	0.06
36	48.74 (10.83)	45.75 (9.95)	2.99 (-1.54 to 7.49)	0.10	

ICC – intra-class correlation coefficient

*Values are means (standard deviation) unless otherwise stated. ICC- intra-class correlation coefficient.

**†**The variation in n was due to missing data because some children did not complete all the tests.

‡Mean differences (95% CI) of Ages and Stages Questionnaire scores.

§*P-*value is for the difference between the two study groups adjusted for clusters.

**Table 4:** Mullen Scales of Early Learning scores obtained in the two study groups at 36 months*

	Intervention (n = 74)	Control (n = 73)	Between group difference†	*P*-value‡
Visual reception	53.31 (13.63)	50.33 (12.44)	2.98 (-7.24 to 1.27)	0.17
Fine motor	62.84 (15.55)	56.18 (14.91)	6.66 (1.69 to 11.83)	0.009
Receptive language	58.72 (10.33)	55.10 (11.26)	3.62 (0.10 to 7.14)	0.044
Expressive language	60.59 (10.33)	56.25 (10.51)	4.34 (0.95 to 7.74)	0.012
Cognitive total score	235.46 (42.27)	217.85 (41.35)	17.61 (3.98 to 31.24)	0.012
Early learning score	75.64 (29.17)	64.77 (31.67)	10.87 (1.81 to 14.87)	0.013

*Values are means (standard deviation) unless otherwise stated.

**†**Mean differences (95% confidence interval) of Mullen Scales of Early Learning scores.

‡*P-value* is for the difference between the two study groups adjusted for clusters.

### Growth outcomes

The mean HAZ declined in both study groups during the study period, indicating linear growth faltering (**Table 5**). However, this decline was significantly less at 36 months in the intervention compared with the control group. There were no significant differences in the other mean anthropometric measures (ie, WAZ, WHZ, and HCZ) at 36 months. The Cohen’s d effect sizes at 36 months were 1.01, 0.16, -0.46, and 0.30 for HAZ, WAZ, WHZ, and HCZ, respectively.

**Table 5:** Child growth during the study period*

Age of child (months)	Intervention (n = 74-77)†	Control (n = 73-78)†	Between group difference‡	*P-v*alue§	ICC
**Height-for-age z-scores:**					
20-24	-1.96 (1.14)	-2.07 (1.20)	0.11 (-0.14 to 0.35)	0.41	0.34
36	-2.15 (1.01)	-2.65 (0.88)	0.50 (0.25 to 0.75)	0.0001	
**Weight-for-age z-scores:**					
20-24	-0.76 (0.88)	-0.85 (0.88)	0.09 (-0.37 to 0.55)	0.70	0.10
36	-0.98 (0.89)	-1.18 (0.69)	0.20 (-0.27 to 0.66)	0.40	
**Weight-for-height z-scores:**					
20-24	0.26 (0.94)	0.45 (0.77)	-0.19 (-0.52 to 0.16	0.31	0.04
36	0.44 (0.91)	0.84 (0.74)	0.40 (-0.75 to 0.05	-0.054	
**Head circumference z-scores:**					
20-24	0.30 (0.93)	0.61 (1.05)	-0.25 (-0.64 to 0.04)	0.079	0.00
36	-0.34 (0.90)	0.05 (1.01)	-0.39 (-0.72 to 0.34)	0.055	

*Values are means (standard deviation) unless otherwise stated. ICC-intra-class correlation coefficient.

**^†^**The variation in n is due to missing data.

‡Mean differences (95% confidence interval).

§*P-*value is for the difference between the two study groups adjusted for clusters.

### Gut microbiota composition

**Figure 2:**
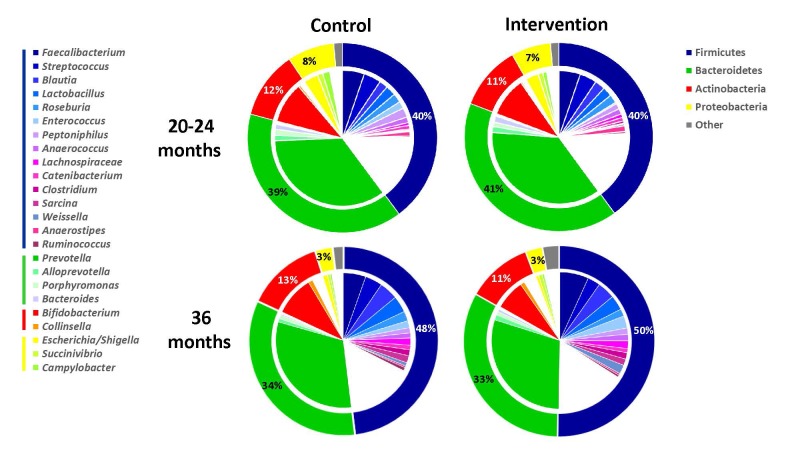
Fecal microbiota compositions based on normalized 16S rRNA amplicon sequencing reads from the control (left pie charts) and intervention (right pie charts) group at 20-24 (upper pie charts) and at 36 (lower pie charts) months. The outer donuts represent the four predominant phyla (legend: right upper corner) and the inner pie charts the most abundant genera within each of these phyla (legend: left). Charts indicate the average relative abundance of phyla and genera in the fecal microbiota of the children with a cut-off value of 0.7%.

**Figure 3:**
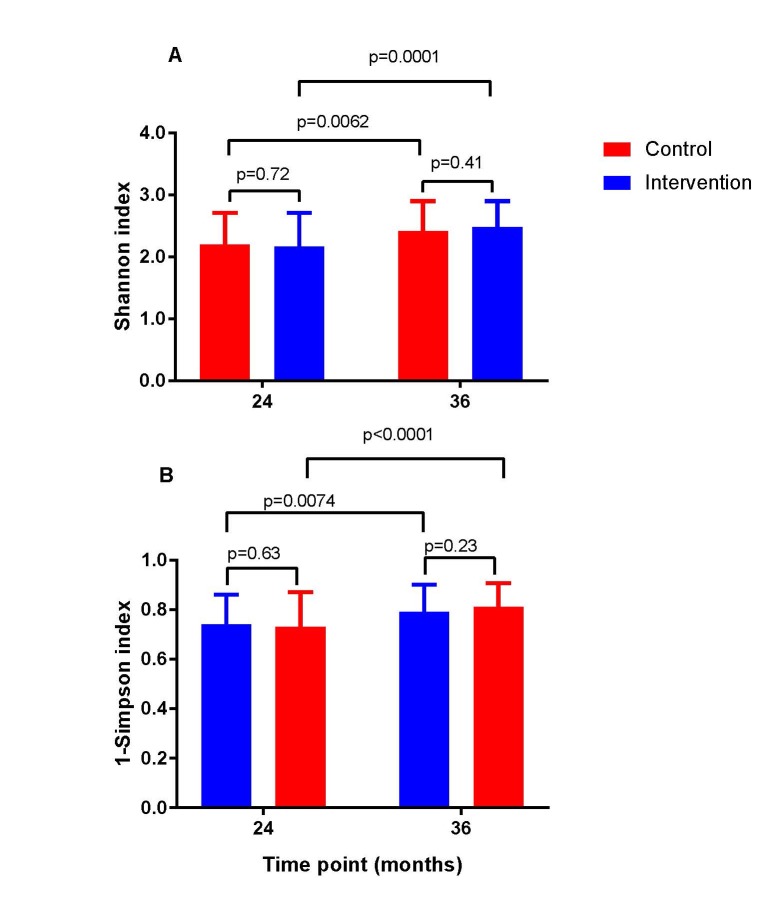
Shannons diversity index (**A**) and 1-Simpsons index (**B**) for gut microbiota diversity obtained from the control (open symbol) and intervention (closed symbol) group among the children at age 20-24 and 36 months. Values are mean ± standard deviation.

The intervention did not lead to any significant changes in the gut microbiota diversity compared with the control group at the phylum level (**Figure 2**). Neither did we observe any significant differences between the two study groups in the Shannon diversity index at the two time points (**Figure 3**). However, as expected the Shannon diversity index increased significantly in both study groups from 20-24 to 36 months, indicating increased gut microbiota diversity, while there was no significant change in the overall genera distribution from 20-24 to 36 months. In line with this, there was no change in the variable 1-Simpson index between the two study groups at the two time points (**Figure 3**), and this variable increased from 20-24 to 36 months, again indicating increased gut microbiota diversity. In support of these findings, the PERMANOVA analysis revealed that there was a significant change in the composition of the gut microbiota from 20-24 to 36 months, both at the genus (*P* = 0.001) and at the phylum (*P* = 0.001) level, but that there was no significant effect (*P* = 1) of the intervention on the overall gut microbiota composition.

## DISCUSSION

This is probably the first randomized education intervention trial incorporating gut microbiota analysis in rural Sub-Saharan Africa. In the parental trial the 6-month education intervention led to significant improvements in development outcomes when the children reached 20-24 months, without affecting growth [15]. We now show a sustained improvement in the development outcomes even at 36 months and with the use of three independent tools. The intervention also reduced linear growth faltering until 36 months, but had no effect on gut microbiota composition.

Our effect sizes on child development outcomes were comparable/higher than those obtained in previous studies that included nutrition supplementation and child stimulation intervention [5,29,30]. Studies from low income countries using the BSID-III to assess development found that children who received both nutrition and responsive stimulation reported higher cognitive, language and motor skills compared to those who only received either nutrition or stimulation [25,31]. Notably, most of these studies provided micronutrient supplementation and play materials whereas we educated the mothers without supporting them with either food or toys. Mother-child play interaction models promote children’s engagement in several activities that enhance development [32]. Moreover, our findings are in accordance with a previous Ugandan study which reported slightly higher cognitive scores three months after stimulation and nutrition education [20].

The baseline stunting levels observed in the two study groups compare favorably with those of previous surveys in Uganda [33]. Corroborating the anthropometric results obtained when the children were 20-24 months [15], most growth indicators were not significantly different between the two study groups at 36 months. The only exception was a smaller reduction in linear growth faltering in the intervention compared with the control group. This could imply that the education intervention may have a protective effect against linear growth faltering over time.

Emerging data suggest links between gut microbiota composition and stunting as well as cognition in childhood [34,35], possibly mediated through cross-talk between microbiota-derived signaling molecules and host tissues [36]. As improved diet and hygienic practices may promote a healthy gut microbiota [37], interventions to enhance nutrition may indirectly impact positively on child growth and development outcomes [38,39]. Previous studies on nutrition and gut microbiota are mostly based on animal models or clinical trials with specific nutrients, pre- or probiotics to modify microbiota diversity [39]. In the present trial we emphasized education of the mothers about preparing nutritious foods, ensuring hygienic meal preparations and maintaining good oral health among their children. Despite acceptable adherence to this intervention [15], we could not detect any significant effects on gut microbiota composition after 20-24 or after 36 months.

Our baseline data on maternal and household characteristics were in line with previous reported data from Uganda [40-42]. Our education intervention consisted of a combined strategy to improve nutrient intakes, hygiene/sanitary practices and stimulation through increased knowledge and empowerment of the mothers. Although it is not possible to exactly specify which component(s) led to the improvement in child development outcomes, the unchanged child diet diversity observed among the households in the intervention group at 20-24 months [15] as well as the unaltered gut microbiota, suggest that the improvements were predominantly resulting from enhanced stimulation and hygiene practices. A systematic review of combined nutrition and stimulation interventions reported that child development was consistently improved through stimulation while growth and nutritional status were usually improved by nutrition [43]. Although this review found little evidence for combined benefits of both nutrition and child stimulation interventions on child development, our findings indicate that having a combination of nutrition, hygiene and child stimulation education may have a potential benefit on child development outcomes.

## Strengths and limitations

In this study we adopted a multidisciplinary approach combining aspects of nutrition, hygiene, psychology, microbiology and validated research instruments. Of note, the children were followed for several years. Despite that only about one-third of the mother-child pairs of the parental trial could be re-enrolled for this follow-up study, the latter cohort was well balanced with the baseline characteristics of the parental cohort. A limitation of our study was lack of baseline data of gut microbiota composition, and we have no information about body composition, dietary intakes or relevant biomarkers among the children or if the mothers in the intervention group continued to stimulate their children in the period between end of intervention and when the children reached the age of three years. ASQ is a maternal report and could possibly be biased. Furthermore, we do not report on maternal mental health which may impact on development and growth of small children, in particular in low-resource settings [44].

## CONCLUSIONS

This nutrition, hygiene and stimulation education intervention among mothers of 6-8 months old children had a positive effect on child development and growth until 36 months. We found no significant effects of the intervention on gut microbiota composition. The positive effects from this intervention would call for further research of such an intervention before consideration of scale-up and implementation in other low-income rural settings.
